# Pregnancy outcomes and associated factors for uterine rupture: an 8 years population-based retrospective study

**DOI:** 10.1186/s12884-022-04415-6

**Published:** 2022-02-01

**Authors:** Sheng Wan, Mengnan Yang, Jindan Pei, Xiaobo Zhao, Chenchen Zhou, Yuelin Wu, Qianqian Sun, Guizhu Wu, Xiaolin Hua

**Affiliations:** 1 Department of Obstetrics, Shanghai First Maternity and infant hospital, Shanghai Tongji University School of Medicine, 2699 West Gaoke Road, Shanghai, 201204 China; 2 Department of Gynecology, Shanghai First Maternity and infant hospital, Shanghai Tongji University School of Medicine, 2699 West Gaoke Road, Shanghai, 201204 China

**Keywords:** Uterine rupture, Cesarean section, VBAC, Maternal and neonatal outcome, Risk factors

## Abstract

**Background:**

Uterine rupture is an obstetrical emergency with serious undesired complications for laboring mothers resulting in fatal maternal and neonatal outcomes. The aim of this study was to assess the incidence of uterine rupture, its association with previous uterine surgery and vaginal birth after caesarean section (VBAC), and the maternal and perinatal implications.

**Methods:**

This is a population-based retrospective study. All pregnant women treated for ruptured uterus in one center between 2013 and 2020 were included. Their information retrieved from the medical records department were reviewed retrospectively.

**Results:**

A total of 209,112 deliveries were included and 41 cases of uterine rupture were identified. The incidence of uterine rupture was 1.96/10000 births. Among the 41 cases, 16 (39.0%) had maternal and fetal complications. There were no maternal deaths secondary to uterine rupture, while perinatal fatality related to uterine rupture was 7.3%. Among all cases, 38 (92.7%) were scarred uterus and 3 (7.3%) were unscarred uterus. The most common cause of uterine rupture was previous cesarean section, while cases with a history of laparoscopic myomectomy were more likely to have serious adverse outcomes, such as fetal death. 24 (59.0%) of the ruptures occurred in anterior lower uterine segment. Changes in Fetal heart rate monitoring were the most reliable signs for rupture.

**Conclusions:**

Incidence of uterine rupture in the study area, Shanghai, China was consistent with developed countries. Further improvements in obstetric care and enhanced collaboration with referring health facilities were needed to ensure maternal and perinatal safety.

**Supplementary Information:**

The online version contains supplementary material available at 10.1186/s12884-022-04415-6.

## Background

Uterine rupture (UR) is a full-thickness separation of the uterine wall through breaching during pregnancy, labor, or immediately after delivery [1–3]. According to the world health organization, the average incidence of UR is 5.3/l0 000 [1]. UR is one of the most dangerous obstetric problems and a life-threatening emergency. It is an important cause of maternal and perinatal morbidity and mortality [4–6]. Maternal mortality ranges between 1 and 13% and neonatal mortality between 74 and 92% as a result of UR [1]. The determinant factors for maternal and fetal outcomes of UR differ across geographical regions due to differences in socio-demographic status, the availability and accessibility of routine obstetric care, and health system effectiveness. Analyzing outcomes and factors associated with maternal and fetal complications of UR in the study area, Shanghai, is important to prevent and improve local clinical management by designing appropriate policies and strategies.

Although the occurrence of UR is relatively rare in general, it is more frequent in low-income compared to high-income countries [7, 8]. In high-income countries, the greatest risk factor is a scarred uterus, typically from a previous cesarean delivery. Risks of UR are also related to other factors, such as parity, obstructed labor, induction of labor, use of prostaglandins, and/or breech presentation [1, 7, 9]. VBAC (vaginal birth after caesarean section) is an important practice to reduce caesarean section rate. However, in China, many hospitals are reluctant to attempt a TOLAC (trial of labor after caesarean delivery) due to increased risks of severe adverse outcomes, such as UR and fetal or neonatal death. Nevertheless, reports on UR and its maternal and perinatal outcomes for such delivery are lacking in China. As to scarred uterus, previous studies on the outcomes of UR were generally concentrated on patients with previous cesarean section, while very few studied patients with other gynecological surgery history.

The aim of this study was to analyze all cases of UR in our hospital during the period 2013–2020 to assess the incidence, its linkage with previous caesarean and other gynecological surgery history, and the maternal and perinatal risk factors as well as the implications of UR.

## Methods

### Study design and participants

A retrospective analysis was conducted using UR cases recorded at the Shanghai First Maternity and Infant Hospital, Tongji University School of Medicine from June 1, 2013 to December 31, 2020. This hospital is a tertiary referral center for critical and severe diseases of pregnant and delivery women and has the largest number of deliveries in the Eastern China region. This study was approved by the Ethics Review Committee of Shanghai First Maternity and Infant Hospital, Tongji University School of Medicine (KS20268). We excluded cases that were pregnancies before 20 weeks or experienced traumatic of motor vehicle accidents.

### Variables of the study

Patients with UR were divided into two groups according to maternal and/or fetal complications or not. A comparison between the two groups were conducted. Maternal complication was defined as the postpartum hemorrhage (a cumulative blood loss of greater than or equal to 1000 mL or blood loss accompanied by signs or symptoms of hypovolemia 24 h post birth) [10], hysterectomy, obstetric injury (genital and/or urinary injury), and maternal death. Neonatal complication was defined as Apgar score < 7 at 5 min, neonatal intensive-care unit (NICU) admission, and neonatal death [11, 12]. A complete UR was defined as tearing in all layers of the uterine wall, including the serosa and amniotic membranes. An incomplete UR was defined as tearing in the muscular layers, with intact serosa or amniotic membranes [13].

We retrieved the charts of UR cases and collected four independent variables: 1) socio-demographic characteristics, including age, parity, education, and place of residence; 2) pregnancy and labor related variables, such as previous cesarean section, ectopic pregnancy, uterine myomectomy and other uterine operation history, and intrauterine operation; 3) clinical symptoms and signs; and 4) maternal and fetal outcomes (delivery method, blood loss and transfusion, postpartum hemorrhage, ICU, birth weight, 5-min Apgar score < 7).

### Data processing and analysis

All collected data were rechecked for completeness and coded. Then the data were entered and processed using Epidata 3.1 software. Data are expressed as mean ± standard deviation, or median (25th–75th percentile). The normality of variables was assessed. Differences between the two groups were compared with the Student’s t-test and the Mann–Whitney U test for continuous variables: mean and median, respectively, and with the χ2 test or Fisher’s exact test for categorical variables. We used the Spearman coefficient to assess the correlation between UR rate and VBAC rate. Multivariable logistic regression analysis was performed to examine the association of included variables with UR. Odds ratios (OR) were presented with 95% confidence intervals (CI). Statistical analyses were performed using SPSS software, version 22.0 (SPSS Inc., Chicago, IL, USA). A *p* value of less than 0.05 was considered statistically significant.

## Results

During the study period, 41 UR were identified among a total of 209,112 deliveries. The incidence of UR was 1.96/10000 births. There were no maternal deaths, hysterectomy, or obstetric injury secondary to UR found in our study. Among all cases, there were 16(39.0%) cases with complication and 25(61.0%) cases without; 15 (36.6%) were complete rupture cases and 26 (63.4%) were incomplete rupture cases; 38(92.7%) were scarred uterus and 3 (7.3%) were unscarred uterus.

**Fig. 1:**
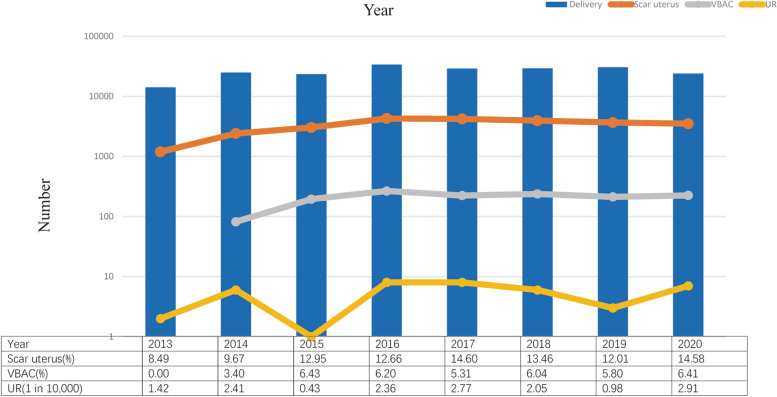
Trend of uterine rupture, scar uterus and VBAC at Shanghai First Maternity and Infant Hospital, 2013–2020. Logarithmic scale is used to indicate the number on the vertical axis

The total number of deliveries and the rates of scarred uterus and VBAC increased over the eight years. However, the proportion of UR remained consistent (Fig. 1). UR rate was not associated with VBAC rate (correlation coefficient: − 0.095, *p* = 0.826).

Demographic data and clinical characteristics of mothers and fetuses between UR and non-UR were presented in Table 1. Patients in UR group were significantly older and more than half (58.5%) of them were over 35 years old, compared to the 18.8% of the non-UR group. The mean gravidity of the case women of the UR group was 2.95 ± 1.41, significantly higher than that of the non-UR group (1.85 ± 1.09). The proportion of primiparity in non-UR group (72.7%) were significantly higher than UR group (24.4%). There was a statistically significant difference in the gestational age at delivery (39.0 ± 1.6 vs. 37.0 ± 3.5), birth weight (3296.9 ± 470.1 vs. 3016.6 ± 755.1) and maternal hospital stay (4.3 ± 4.1 vs 7.7 ± 5.3) between the groups (*p* < 0.05). Compared to the non-UR group, the proportion of gestational hypertension (7.3% vs. 1.1%), artificial reproductive technology (12.2% vs. 4.0%), cesarean delivery (100.0% vs 39.9%), postpartum hemorrhage (31.7% vs. 1.5%), preterm birth (39.0% vs. 6.6%), and 5-min Apgar score < 7 (19.5% vs. 1.0%) were significantly higher in the UR group (*p* < 0.05).

**Table 1:** Characteristics of mothers and newborns in study

	Non-UR	UR	***p*** value
**Mothers**			
Age (years)	30.9 ± 4.0	35 ± 3.78	< 0.001^*^
> 35 y	39,313 [18.8]	24 [58.5]	< 0.001^*^
Gravidity	1.85 ± 1.09	2.95 ± 1.413	< 0.001^*^
Primiparity	152,024[72.7]	10[24.4]	< 0.001^*^
Gestational diabetes mellitus	22,793[10.9]	6[14.6]	0.605
Gestational hypertension	2300[1.1]	3[7.3]	0.002^*^
Artificial reproductive technology	8365 [4.0]	5[12.2]	0.023^*^
Hospital stay	4.30 ± 4.10	7.71 ± 5.28	< 0.001^*^
Postpartum hemorrhage	3137 [1.5]	13[31.7]	< 0.001^*^
**Deliveries/Newborns**			
Cesarean delivery	83,436[39.9]	41 [100]	< 0.001^*^
Gestational age (weeks)	39.00 ± 1.60	37.04 ± 3.52	0.001^*^
Preterm birth (< 37 weeks)	13,801 [6.6]	16[39.0]	< 0.001^*^
Birth weight (g)	3296.9 ± 470.1	3016.59 ± 755.1	0.022^*^
Macrosomia	11,083 [5.3]	1[2.4]	0.639
5 min Apgar< 7	2091 [1.0]	8[19.5]	< 0.001^*^

*p<0.05, values are expressed as mean ± standard deviation or number [percentage]

*UR* Uterine rupture

Table 2 displayed the occurrence of obstetrical risk factors in complicated or not complicated UR groups. Among all patients with UR, 16 (39.0%) had maternal and fetal complications. Postpartum hemorrhage was the main maternal complication and most blood loss occurred during the surgery. 13 cases had blood loss above 1000 ml. Among them, five had excessive blood loss above 2000 ml. As to fetal complications, we had 8 cases of Apgar score < 7 at 5 min, 5 cases of NICU admission, and 3 cases of neonatal death. Compared with not complicated UR, women in complicated UR group had higher proportions of primiparity, uterine myomectomy history, artificial reproductive technology use, blood transfusion, intensive care unit (ICU) admission, and complete UR. Complicated UR group also presented a longer hospital stay, a higher probability of preterm birth, multiple pregnancy, a smaller rupture gestational weeks, a lower birth weight, and prevalence of previous cesarean history.

**Table 2:** Characteristics of mothers and newborns in complicated and not complicated uterine rupture

	Complicated	Not complicated	***p*** value
	16	25	/
**Mothers**			
Age (years)	35.77 ± 4.38	34.56 ± 3.64	0.357
> 35 y	10[62.5]	14[56]	0.680
Gravidity	3(1.5–4)	3(2–3.5)	0.517
Primiparity	8[50]	2[8]	0.002^*^
Intrauterine operation	10[62.5]	12[48]	0.364
Gestational diabetes mellitus	2[12.5]	4[16]	0.757
Gestational hypertension	3[18.75]	0[0]	0.053
Artificial reproductive technology	4[25]	1[4]	0.045^*^
Scarred uterus	13[81.25]	25[100]	0.053
Previous cesarean	6[37.5]	22[88]	0.001^*^
Previous UM	5[31.25]	1[4]	0.016^*^
Previous cornual pregnancy	3[18.75]	2[8]	0.305
TOLAC	2[12.5]	8[32]	0.156
Rupture of GA	36.14(30.86–37.86)	38.71(37.43–39.79)	0.001^*^
Interval since last operation (years)	4(2.5–6.5)	4(3–6.5)	0.584
Diagnosed in surgery	10[62.5]	10[40]	0.16
Transfusion	8[50]	1[4]	0.001^*^
Intensive care unit	11[68.75]	1[4]	<0.001^*^
Hospital stay (days)	7(5–10.5)	5(4–7)	0.043^*^
Abnormal fetal heart rate	11[68.75]	6[24]	0.005^*^
Vaginal bleeding	7[43.75]	6[24]	0.007^*^
Abdominal pain	11[68.75]	12[48]	0.192
Other symptoms	0[0]	5[20]	0.137
Emergency indication	13[81.25]	14[56]	0.096
Complete UR	9[56.25]	6[24]	0.036^*^
**Deliveries/Newborns**			
Preterm birth (< 37 weeks)	10[62.5]	6[24]	0.014^*^
Twins	4[25]	0[0]	0.018^*^
Birth weight (g)	2970(1740–3500)	3200(2945–3635)	0.040^*^

*p<0.05, values are expressed as mean ± standard deviation, number [percentage], or median (Q1–Q3)

*UM* uterine myomectomy, *TOLAC* trial of labour after caesarean delivery, *GA* gestational age, *UR* uterine rupture, *NICU* neonatal intensive care unit

Patients’ rate of abnormal fetal heart rate (68.8% vs. 24.0%) and vaginal bleeding (43.8% vs. 24.0%) were significantly higher in the UR group with maternal and fetal complications. Among all 16 cases of complicated UR, eight cases presented signs and symptoms during pregnancy, five cases with the onset of labor and three cases during the process of labor. In the complicated group, the range of ruptured gestational week was 23 to 40 weeks. In the not complicated group, the earliest and the latest ruptured gestational week were 35 weeks and 40 weeks respectively. No maternal death was observed. The perinatal fatality attributable to UR was 7.3%. 21 (51.2%) mothers were diagnosed with UR preoperatively and 20 (48.8%) were diagnosed intraoperatively. The diagnosed time and the proportion of TOLAC were similar in the 2 groups (*p* = 0.156).

Multiple logistic regression analyses were employed to examine whether signs and symptoms were associated with the presence of UR with complication (Table 3). The model, which included all signs and symptoms as independent variables, showed that the abnormal fetal heart rate emerged as a significant and independent factor associated with the complicated UR compared with other signs. (OR = 12.45; 95% CI: 1.16–133.54; *p* < 0.05). Other clinical signs were not statistically different. All three cases of neonatal deaths presented abdominal pain while two of them presented abnormal fetal heart rate at the same time. We also showed their detailed descriptions in Supplementary Table 1.

**Table 3:** Signs and symptoms of rupture uterus presented in a multi-variable analysis

	OR	95%CI		***p*** value
Abnormal fetal heart rate	12.446	1.16	133.54	0.037^*^
Vaginal bleeding	0.807	0.055	11.932	0.876
Abdominal pain	2.062	0.356	2.062	0.419
Other symptoms	0	0	/	0.999

*p<0.05, *OR* odd risk, *CI* Confidence intervals

**Fig. 2:**
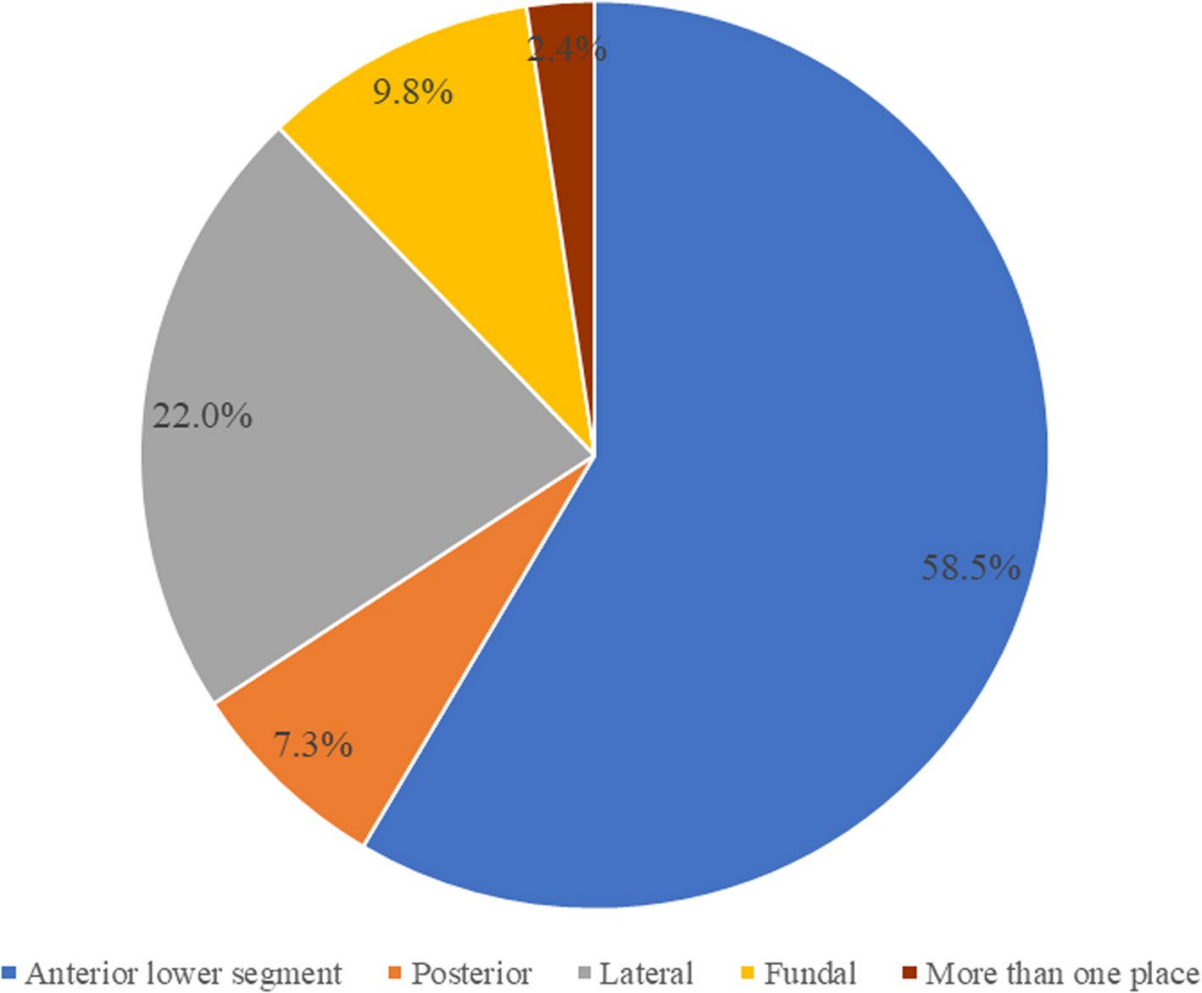
Site of uterine rupture

Figure 2 shows the rupture sites involved. 24 (58.5%) cases were anterior lower uterine segment; 3 (7.3%) cases had posterior segment rupture; 9 (22.0%) cases were ruptured at the lateral segment; and 4 (9.8%) cases were fundal segment rupture and one ruptured more than one place (2.4%).

Detailed clinical information on all UR cases following laparoscopic myomectomy is shown in Table 4.

**Table 4:** Detailed surgical findings and obstetric outcomes of the six cases with uterine rupture following laparoscopic myomectomy

Patient	1	2	3	4	5	6
Age(yr)	30	39	44	33	37	32
Year of surgery	2014	2007	2013	2015	2018	2016
Number of myoma removed	5	1	2	2	2	2
Myoma type	IM	IM	IM, SS	IM	IM, SS	IM
Myoma size(cm)	6,3*4	6	5,1.5	3*2	6,1	6,2
Uterine incision	MP	MP	MP	MP	MP	MP
Cavity entered	No	No	No	No	No	No
Hemostasis type	BP, S	BP, S	BP, S	BP, S	BP, S	BP, S
Stitches	3 Layers	2 Layers	2 Layers	2 Layers	2 Layers	2 Layers
Anti-adhesion agents	No	No	No	DM	DM	Yes
Interval from surgery to pregnancy(yr)	2	9	5	3	2	4
Gestational week of rupture	31.43	36.43	37.43	30.29	23	35.43
Labor	No	No	No	No	No	No
Volume of bleeding(ml)	3250	800	2000	2500	2850	1250
Number of fetuses	1	1	1	1	1	1
Fetal survival	No	Yes	Yes	No	No	Yes
Maternal survival	Yes	Yes	Yes	Yes	Yes	Yes

*p<0.05, *BP* bipolar electrosurgery, *DM* data missing, *IM* intramural, *MP* monopolar electrosurgery, *S* suture, *SS* subserosal

## Discussion

UR in pregnancy is rare, but when it occurs the consequences can be life-threatening to both the mother and the fetus [14, 15]. The occurrence of UR varies in different parts of the world. Globally, the incidence of UR is 0.07% with the tendency of being lower in the developed countries than the developing countries [1, 16]. The rate of UR in our study was 0.0196%, consistent with the rate observed in the developed countries as Shanghai is among the most economically developed regions in China, which is close to developed countries. There were no cases of maternal death due to UR in our study.

There has been a wide variation in the aetiology UR over years [17–19], where the increase rate of TOLAC and the use of uterotonics have created the two most common predisposing factors in the developed countries [9, 16, 20, 21]. However, the major causes of UR in the developing countries are both obstetric and non-obstetric multitude of factors: multi-gravidity, teen-age pregnancy, old primi, poor socio-economic status, previous cesarean section scar, unsupervised labor, and unwise use of uterotonic agents [4].

Our study showed that the key risk factor of UR was the presence of scar, and previous cesarean section is the most important cause of uterine scarring. Therefore, to reduce UR rate, we need to strictly control the indication of cesarean section so as to reduce the rate of cesarean section. Globally, cesarean delivery rates have been steadily increasing over the past 20 to 30 years [22–24]. A major contributor to this has been elective repeat cesarean sections. Approximately one-third to half of the elective cesareans are performed because of a history of cesarean delivery [22, 25, 26]. Routine elective repeat cesarean section for all women with a prior cesarean section is not universally advocated, desired, or without risk. Furthermore, multiple cesarean sections also carry the increased risks of placenta previa and placenta accrete with future pregnancies [27]. Such a policy would result in significant financial cost [28]. On the other hand, VBAC is able to resolve such problems. As another mode of birth after caesarean section, VBAC is associated with fewer complications, such as shorter maternal hospitalization, less blood loss, and a decreased incidence of puerperal infections and thrombotic events [29]. TOLAC is a safe option for most people and 75% women may be successful [30]. In recent years, VBAC has been supported as a way to decrease related complications and slow the increase in cesarean births to some extents. For instance, in Norway, all mothers with a previous caesarean section are offered a chance of TOLAC unless there is an absolute contra-indication. As a result, the TOLAC rate is as high as 51, and 80% succeed in that country [31]. While VBAC is being advocated by more and more countries, the VBAC rate in China was only 9.6% in 2016, compared to 12.4% in the United States in the same year [32, 33]. While TOLAC is an accepted practice in hospitals with advanced medical equipment and obstetric skills, it still can be controversial. A successful VBAC is associated with fewer complications compared with elective repeat cesarean delivery, whereas a failed TOLAC is associated with more complications [34]. TOLAC has gone through three stages in the U.S. Stage one, the VBAC rate had increased from 5% in 1985 to 28.3% by 1996 as recommendations favored TOLAC; Stage two, the VBAC rate had decreased to 8.5% by 2006 as the number of UR and other complications related to TOLAC increased. During that time, some hospitals stopped offering TOLAC altogether; Stage three, VBACs had been on the rise again since 2016 and increased to 13.3% by 2018, when a balance between TOLAC and safety was reached [33, 34]. The U.S. experience is worth learning and most part of China is currently going through the stage two, so we can see the reversal of the VBAC. Therefore, promoting TOLAC in China and ensuring safety is needed. In our study, we were expecting UR rates to become higher as more people attempted a TOLAC. However, this was not the case observed from this study and ruptures occurring after TOLAC did not become more serious. The ACOG (American College of Obstetricians and Gynecologists) recommended TOLAC depending on the hospital’s resources and availability of obstetric, pediatric, anesthesiology, and operating room staffs [34]. Our hospital is one of the three hospitals with the largest number of births in China, and Shanghai is among the top medical treatment areas in China, which is close to developed countries. Therefore, we have rich enough medical experience to reduce the occurrence of UR and ensure the maternal and perinatal safety. Our study provides evidence that under the condition of strict control and indication, TOLAC is safe and reliable and worth carrying out. With the implementation of birth encouragement policy in China, an increasing amount of second-child pregnant women are choosing to attempt a TOLAC. As a result, the rate of cesarean section and the consequent risks of UR will decline as a whole, and the national medical burden and financial expenditure can be reduced.

The other two causes of uterine scarring identified in our study are previous myomectomy and previous cornual pregnancy. All cases with a previous myomectomy surgery were performed by laparoscopy. With the rise of minimally invasive techniques, laparoscopic surgeries are being performed in greater numbers today than ever before. Despite the overwhelming evidence that laparoscopic myomectomy is minimally invasive and associated with fewer perioperative complications, there is one question that is still under debate – does laparoscopic myomectomy increase the risk of subsequent UR? While some previous studies showed that there was no difference between laparoscopic and open myomectomy on the risk of UR, others demonstrated that laparoscopic procedure increased UR risk compared to open approach because it was believed to result in incompletely repaired muscle defects [35–38]. The use of powered instruments, limited instrumentation use, and the impossibility of palpation might be the reasons. Some techniques including multi-layer closure of the myometrium and limited use of electrosurgical energy should be adhered to by surgeons to decrease the risk [38]. In our study, it seems to lead to more serious outcomes regarding the six UR cases following laparoscopic myomectomy. Among them, four had excessive blood loss above 2000 ml and presented signs of hemorrhagic shock, three of which had the worst outcome, i.e., the fetuses did not survive. The patients might even be influenced by long-term sequelae, which can adversely affect subsequent pregnancies. The removed myoma size and number in UR patients were within the average range of normal cases of laparoscopic myomectomy, which is consistent with other studies [38, 39]. In addition, there is no evidence that indicates the appropriate length of contraception period needed after myomectomy to avoid UR. Currently this interval varies by facility [35]. Some suggested that 12 months might be adequate while others concluded there was no safe interval [35, 39, 40]. In our study, the only UR case without serious complication after laparoscopic myomectomy had an interval of nine years, which is the longest among the six cases. This finding indicates that keeping the duration of the contraception period longer will be safer for patients with a history of laparoscopic myomectomy. Therefore, clinicians must remain vigilant, particularly when the patients have a history of laparoscopic myomectomy. Regardless of the cause of scar uterus, special monitoring is needed during pregnancy and childbirth to ensure the health of the mother and the newborn.

In contrast to UR in women attempting TOLAC, the UR in women with unscarred uteruses occurs often completely unexpectedly. We found an incidence of UR among women who did not have previous uterine scar was 3/209112 deliveries, which was in agreement of the incidence found by Thisted et al. using data from the Danish Medical Birth Registry [21]. All three UR cases in our study that were uncompleted UR found during the cesarean section with almost the same maternal and fetal complications rates as scarred uterus. Among them, two (2/3) were multiple pregnancies with uterus contraction before the cesarean section, and one fell to birth vaginally because of obstructed labor. Our findings suggested that multiple pregnancies and obstructed labor are two major risk factors for UR in patients without a history of previous uterus surgery, which is in line with the recent reports published by Gibbins et al., Vandenberghe et al. and Vilchez et al. [41–43].

Timely detection of UR is conducive to improving maternal and infant outcomes. Symptoms are the only indicators that change dynamically, which can provide the first-hand information for the doctors. In the past, caregivers were taught to look for classic signs such as sudden tearing uterine pain, vaginal hemorrhage, cessation of uterine contractions, Bandl’s ring, and regression of the fetus [44, 45]. However, some studies have shown that these signs are not specific and are often absent [44, 46]. Our study shows that the change of the fetal heart rate is the most reliable presenting clinical symptom. Most of the cases also presented with abnormal pain and vaginal bleeding. Alertness to these signs is the key to the timely rescue and successful management. Other studies had the same conclusions consistent with ours [44, 46].

The most common site of rupture was in the lower uterine segment (58.5%) in our study, which was the scar site of the previous cesarean section. This result is consistent with the findings of the study done by Rizwan et al. [4], in which 80% of the rupture was observed in the lower uterine segment.

Our study has several strengths: (1) a population-based single-centered study, (2) covering a long period of time between 2013 and 2020 with a large sample size, (3) because all patients were delivered in a medical institution, we have a complete and systematic review of all medical records. All patients were followed up six weeks after delivery and no serious complications were found after discharge. However, the study is limited to Shanghai subjects and has limitations owing to the retrospective design. It only represents the level of developed regions in China. The situation in other parts of china is still unknown; thus, further research is needed to understand the generalizability of the study findings.

In conclusion, UR is a disastrous and fatal event for obstetricians and patients. In order to reduce maternal and infant mortality, obstetricians should give enough attention to the pregnant women with high risk factors by strengthening the monitoring. TOLAC is a safe and worth promoting type of delivery for the patients, which still has a long way to go in Shanghai and China.

## Supplementary Information

**Additional file 1: Supplementary Table 1**. Detailed descriptions of the three cases with uterine rupture of neonatal deaths.

## Data Availability

The datasets generated during and analyzed during the current study are not publicly available due to privacy concerns but are available from the corresponding author on reasonable request. Prospective scientists who are interested in are welcomed to contact the corresponding author via e-mail.

## Abbreviations

CI: Confidence intervals
ICU: Intensive-care unit
NICU: Neonatal intensive-care unit
OR: Odds ratios
TOLAC: Trial of labour after cesarean delivery
UR: Uterine rupture
VBAC: Vaginal birth after a caesarean section
