# Supplementary material for: Pregnancy outcomes and associated factors for uterine rupture: an 8 years population-based retrospective study
Source: BMC Pregnancy Childbirth. 2022 Feb 1;22:91. doi: 10.1186/s12884-022-04415-6 (PMC8805328; doi:10.1186/s12884-022-04415-6)
Supplement: Supplementary file 1 — Additional file 1: Supplementary Table 1. Detailed descriptions of the three cases with uterine rupture of neonatal deaths. [file 12884_2022_4415_MOESM1_ESM.docx]

**Supplementary table 1.** Detailed descriptions of the three cases with uterine rupture of neonatal deaths.

| Case | 1 | 2 | 3 |
| --- | --- | --- | --- |
| Age | 30 | 37 | 33 |
| Gravidity | 1 | 1 | 1 |
| Primiparity | 0 | 0 | 0 |
| Gestation weeks | 31.43 | 23 | 30.29 |
| Signs and symptoms | Abdominal pain for 2 days, Abnormal fetal heart rate | Abdominal pain for 3 hours | Abdominal pain for a few hours, Abnormal fetal heart rate |
| Complete UR | Yes | Yes | Yes |
| Birth Weight (grams) | 2010 | 570 | 1345 |
| Possible risk factors | history of LM 2 years previous | history of LM 2 years previous,  >35y | history of LM 3 years previous |
| Other characteristics |  | IVF, Intrauterine operation | FGR during pregnancy |

UR, Uterine rupture; LM, laparoscopic myomectomy; IVF, In vitro fertilization; FGR, fetal growth restriction
